# Demand-Driven Data Acquisition for Large Scale Fleets

**DOI:** 10.3390/s21217190

**Published:** 2021-10-29

**Authors:** Philip Matesanz, Timo Graen, Andrea Fiege, Michael Nolting, Wolfgang Nejdl

**Affiliations:** 1 Volkswagen Group, 30163 Hannover, Germany; timo.graen@volkswagen.de (T.G.); andrea.fiege@volkswagen.de (A.F.); michael.nolting@volkswagen.de (M.N.); 2 L3S Research Center, Leibniz University Hannover, 30167 Hannover, Germany; nejdl@l3s.de; 3 Faculty of Electrical Engineering and Computer Science, Leibniz University Hannover, 30167 Hannover, Germany

**Keywords:** sensor-data acquisition, connected vehicles, big data, cloud computing, floating car data, data streaming, fault-tolerant systems

## Abstract

Automakers manage vast fleets of connected vehicles and face an ever-increasing demand for their sensor readings. This demand originates from many stakeholders, each potentially requiring different sensors from different vehicles. Currently, this demand remains largely unfulfilled due to a lack of systems that can handle such diverse demands efficiently. Vehicles are usually passive participants in data acquisition, each continuously reading and transmitting the same static set of sensors. However, in a multi-tenant setup with diverse data demands, each vehicle potentially needs to provide different data instead. We present a system that performs such vehicle-specific minimization of data acquisition by mapping individual data demands to individual vehicles. We collect personal data only after prior consent and fulfill the requirements of the GDPR. Non-personal data can be collected by directly addressing individual vehicles. The system consists of a software component natively integrated with a major automaker’s vehicle platform and a cloud platform brokering access to acquired data. Sensor readings are either provided via near real-time streaming or as recorded trip files that provide specific consistency guarantees. A performance evaluation with over 200,000 simulated vehicles has shown that our system can increase server capacity on-demand and process streaming data within 269 ms on average during peak load. The resulting architecture can be used by other automakers or operators of large sensor networks. Native vehicle integration is not mandatory; the architecture can also be used with retrofitted hardware such as OBD readers.

## 1. Introduction

Modern cars have the potential to be utilized as a globally distributed sensor platform. The data generated, thus, provides insight into the vehicle, its driver, and the vehicle environment. However, these data streams are currently only accessible to the automakers and a limited set of suppliers. The associated potential remains untapped while there is great demand from the private and public sectors.

Insurance companies are part of the interested parties. They require fine-grained vehicle sensor data to provide Usage-Based Insurances (UBI), prevent fraud, and reconstruct the course of accidents. It is a growth market inhibited by the data collection capabilities of current connected car fleets. Most companies track UBI policies through retrofitted hardware solutions that are installed to the vehicles at high-cost [1]. Receiving data directly from the automaker would reduce operational cost significantly [2].

Besides the commercial demand identified in the insurance market, academia also has many active fields of research that depend on vehicle data. Among these fields are driver behavior identification [3], inference of lane change intentions [4], or drowsiness detection [5]. In addition, access to vehicle data is essential for municipalities to facilitate the transition to Smart Cities [6]. For example, cities may utilize vehicle data to reduce fuel consumption of public transportation [7] and predict urban traffic flows from floating car data [8]. In-vehicle sensors also enable the detection of potholes [9], slippery road conditions [10], or emission hot-spots [11]. Each of these examples requires recent data from a large number of vehicles to unfold its full potential. Individual vehicles may not provide sufficient statistical significance and only provide limited geographic coverage.

It is up to the automakers to tap into this potential. They manage vast fleets with millions of connected vehicles. Opening the associated data can thus be accomplished by performing a software update remotely. When designing scalable data platforms, all car manufacturers face the same significant problems:

- Local data privacy laws cause a globally scattered regulatory landscape. The European General Data Protection Regulation (GDPR) is especially strict among the different data privacy regulations. Complying with it, therefore, supports the integration of weaker frameworks. The GDPR requires obtaining informed consent from the data subjects before their Personally Identifiable Information (PII) can be collected [12]. Additionally, GDPR key principles such as data minimization or storage limitation must be applied [13].
- System-design issues are arising from a large number of cars and their diverse sensing capabilities. The potential supply of data exceeds the demand many times over. Typical data consumers only access a subset of the whole fleet, and from these vehicles, only a limited number of sensors are required for their use cases. Consequently, each data consumer may access individual subsets of the total sensing capacity. The system must map this access pattern efficiently. Nevertheless, these subsets can be of substantial size and place high requirements on the scaling properties of the system.
- Even for a single automaker, hundreds of vehicle models are regularly customized with model derivatives for specific regions and local markets. Each can have different sensing capabilities on its own. Additionally, the customers can select optional features that might introduce additional sensors. The sensors themselves might be supplied from different producers with varying data-access channels. Therefore, in addition to the legal heterogeneity, the individual cars are highly heterogeneous.

This paper presents a system that allows individual data consumers to access sensor data from the globally distributed fleets of major automakers. For this purpose, we integrate software directly into the vehicle, and a cloud platform facilitates access to the consumers. This access is either provided via near real-time streaming or recorded trip files. The overall system was natively integrated with a current vehicle platform of the Volkswagen Group and thus provides insights into the capabilities of current connected cars. However, native integration is not mandatory; the software component can also run on hardware retrofitted in the vehicle.

Our manuscript presents a system architecture and its implementation capable of addressing all three connected car challenges described above. No vehicle transmits more than necessary to satisfy the demands of its relevant data consumers. We can enforce GDPR compliance by requiring prior consent to any transmission of personal data and address individual vehicles directly to acquire non-personal data. An abstraction layer enables us to operate in a highly heterogeneous environment that includes cars with different sensor availabilities and technologies. We compress transmitted data by combining a custom data preprocessing strategy with an existing generalized compression algorithm. The system is capable of handling more than 200,000 simultaneously active vehicles. During a performance evaluation, it had consistently low response times after self-adjusting the number of utilized servers.

### Contributions

The main contributions of this paper are as follows:

- A method to minimize the scope of acquired sensor data for each vehicle individually: Each vehicle only transmits data required to fulfill the *demand* relevant to it. A data consumer’s *demand* is considered relevant if the data subject has consented to fulfill it (personal data) or the individual vehicle was assigned to the *demand* (non-personal data).
- Dynamic *demand* determination in the context of a fixed purpose and data scope. For this purpose, the data consumers manage tasks which may also include constraints evaluated on each vehicle locally and data processing options.
- An abstraction layer enables the integration of a heterogeneous fleet through a single interface. For this purpose, we continuously distribute instructions to the vehicles. Each instruction describes an individual sensor access pattern. The vehicle checks these instructions by applying a trial-and-error scheme to determine its sensor capabilities and accessing the corresponding data.
- A compression strategy combines custom data preprocessing with an existing algorithm and, in combination, produces better results than the preprocessing itself or other existing algorithms alone. The compression ratio was evaluated by using actual vehicle data.
- A cloud-based reference implementation that handles data processing as distributed and trip-related transactions. Overall system throughput is automatically adjusted by adding or removing servers.
- Validation of the scaling properties of the overall system through a realistic simulation of over 200,000 simultaneously active vehicles.

The remainder of this manuscript is organized as follows: We present an overview of existing architectures and systems in Section 2. Then, we introduce our proposed system in Section 3 and address implementation details of its main components in Section 4. The results of our performance evaluation are detailed in Section 5. We compare our contributions with the identified works in Section 6. The final Section 7 provides conclusions to the paper.

## 2. Related Work

This section differentiates between works from academia and commercial systems for collecting and distributing vehicle sensor data. We present an in-depth comparison with our contributions in Section 6. In addition, we compare closely related systems with ours in Table 1.

### 2.1. Academia

The development of systems to collect vehicle data is often a prerequisite for answering research questions based on the data obtained [16,17,18,19,20,21]. For example, the authors of [16] have developed driving behavior analysis methods based on continuous streams of vehicular data. For this purpose, they had to develop an infrastructure capable of providing the required data. Such a pattern is indicative of a lack of suitable public data-access channels provided by the automakers.

Customer feedback platforms in the context of the Industry 4.0 vision represent a subset of these specialized data acquisition systems. Such platforms enable manufacturers to receive usage data directly from their products to improve quality, flexibility and productivity [21]. The data obtained in this way usually does not leave the sphere of the automobile manufacturer.

Some works focus exclusively on building platforms to make vehicle data available to arbitrary data consumers [22,23]. In contrast to the previous works, they provide the functionality to handle multi-tenant setups in which distinct parties acquire data from a fleet. For this purpose, they have implemented an authorization layer that restricts data access for the individual tenants: The driver must grant permission for each tenant to access data collected from his vehicle. Permission differs from consent because it does not influence data collection; it takes place in any case.

We utilize consent instead and combine our multi-tenant capabilities with a dynamic procedure to reduce data acquisition for each vehicle individually. For this purpose, we natively integrate software with an existing vehicle platform of the Volkswagen Group. This software selects the sensors to be recorded and can evaluate conditions that influence data acquisition. The sensor selection depends on the sensors required by the data consumers and can be continuously adjusted even after the data subject gave consent. In other works, data acquisition takes place as a static routine that is decoupled from the requirements of individual data consumers [16,18,19,20,21,22,23,24,25,30,31,32].

Apart from [23], the presented systems are not integrated natively with the vehicle. Consequently, the authors had to utilize the On-Board Diagnostics (OBD) interface to acquire sensor data from the vehicle [16,17,18,20,21,22,25]. The OBD is a physical interface within the car that exposes a small fraction of the data available on the Controller Area Network (CAN) [40]. Most sensors are connected to the CAN and broadcast messages containing their latest measurements. The majority of these messages are encoded using a manufacturer’s proprietary scheme, which can also vary depending on the vehicle model [26,40]. Thus, parties unaffiliated with the automaker must reverse engineer the CAN to process all measurements, which is a dedicated field of research [41,42].

While some works use OBD as a sole vehicular data source [16,18,20,21], others also obtain data from their own sensors [17,22,25,43] or use them exclusively [19]. A combination of different data sources can also occur on a higher level by aggregating data from existing telematics systems and smartphones [24]. In some cases, the connection with the OBD is set up via self-designed hardware [44]. Our system is natively integrated with the vehicle via software and does not need additional hardware such as an OBD dongle. It only utilizes sensors that are part of the vehicle and acquires their readings via multiple software-based data-access channels provided by the vehicle platform.

The OBD interface essentially represents an abstraction layer, as it can provide a subset of the overall sensor readings of a vehicle through standardized message formats. Furthermore, it supports the discovery of available standardized sensor readings [21]. We have developed a higher-level abstraction layer that extends these features. It enables the data consumers to influence data acquisition in the absence of specific sensors. Instead of normalizing the sensor readings, they are supplemented with metadata such as resolution or sample rate. In addition, we can connect multiple data-access channels and handle vehicle-specific incompatibilities caused by diverging implementations of the data-access channels.

The acquired sensor data represent time series. There are compression algorithms optimized for time-series data that are not specific to the automotive domain [45,46]. Furthermore, there are generalized compression algorithms that can handle arbitrary binary data [47,48,49,50,51,52,53]. We have created a domain-specific and lossless data preprocessing strategy and combined it with an existing generalized compression algorithm. In [23], the authors utilize downsampling and histograms to reduce data transmission size, and a trajectory compression algorithm is used by [18]. In contrast, the authors of [54] propose to detect anomalies locally on the vehicle and restrict data transmission to them.

Different concepts are used for the processing and storage of data. Some systems provide a REST interface to access stored data requiring the data consumer to query for updates [22,23]. In [23,27], the authors use a message broker to push incoming data directly to data consumers. The works of [16,18] utilize multiple storage layers optimized for specific use cases. For example, the authors of [18] store the most frequently accessed data in a memory-based caching system, use a relational database for preprocessed data, and archive raw data in a NoSQL-based system. Our cloud-native system utilizes a single storage layer that enables near real-time streaming and continuous sensor data recording. We can integrate third-party systems from different clouds and deliver events to them, thus eliminating the need to query for updates.

There are also architectural concepts for acquiring vehicular data [26,27,28,29,55]. These concepts imply an immediate integration into the car without providing a reference implementation. Unlike the previously mentioned works, these architectures are not forced to adapt to the reality of the volatile and complex automotive domain. On the contrary, our work demonstrates the capabilities of modern mainstream vehicle platforms through its integration into a current platform of the Volkswagen Group.

### 2.2. Commercial Systems

There exists a commercial market for systems that enable data acquisition from vehicle sensors. We present a selection and distinguish between software-based solutions and those that require retrofitted hardware installed on the vehicles. These systems are mainly black boxes, and their implementation details are only known to the manufacturers. The descriptions are based on the manufacturers’ public documentations.

#### 2.2.1. Software Platforms

Otonomo [33], Caruso [34] and Smartcar [35] are neutral platforms that act as intermediaries between automakers and data consumers. The data consumer maintains a relationship with the neutral platform, which acquires data from the automaker and forwards it to the customer. The vehicle owner must grant consent by completing an OAuth flow with the automaker as specified by Extended Vehicle Standard [56]. Each of these platforms has a REST interface that provides sensor values of individual vehicles. In addition, they can stream incoming vehicle data to a data consumer supplied server by utilizing HTTP requests. The sensor availability differs across these platforms. Otonomo provides filters that limit the streaming of already collected data based on the location of the vehicles or the age of the data.

Additionally, various car manufacturers offer direct access to vehicle data. These include Mercedes Benz [36], BMW [37], or Ford [38]. They also require the vehicle owner to grant permission for data acquisition by performing an OAuth flow. Streaming is not supported; the customer must poll the data instead.

The Groupe PSA [39] has created a system for acquiring data from fleets belonging to a single owner. Customers can create *Monitors*. They consist of a condition validated on the backend and, if matched, results in a notification to customers. The condition refers either to a geographical area, the time, or an arbitrary vehicle state (e.g., AC active/inactive). Additionally, a record is created for each performed trip. It contains the distance and the start and end position.

#### 2.2.2. Hardware/Software Platforms

Providers such as Zubie [30], Munic [32] or Vinli [31] offer OBD dongles that have built-in internet connectivity and send vehicle data to an associated cloud platform. All systems offer interfaces to poll sensor data from the cloud. Munic and Vinli store multiple updates of a sensor value, while Zubie provides only the last value. Sensor data can only be streamed to the customer via Munic. The others can send events like the start of a trip or its completion. Vinli can validate a condition and trigger an event once it is met.

There are also OBD devices designed to be used as software platforms [14,15,57,58]. As such, their users can deploy custom software to them in order to process and transmit the obtained data. For this purpose, the devices can provide cellular, WiFi, or Bluetooth connectivity. In addition to OBD, some devices also include their own sensors such as GPS or accelerometer [14,15,57]. Some providers also offer software to collect data from their hardware [15] or provide data acquisition as a service via a cloud platform [14,59]. These devices can also be utilized to run the software component we have natively integrated into the vehicle.

## 3. Proposed System

**Figure 1:**
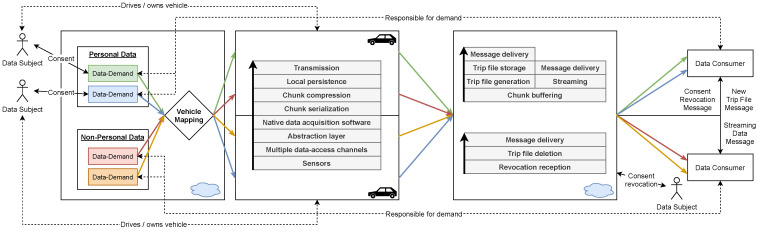
Use of our system to acquire personal and non-personal data.

We have created a system that enables individual data consumers to access the sensing capabilities of vast heterogeneous fleets. It consists of an application that is natively executed by the associated vehicles and a cloud platform. The in-car application includes an abstraction layer that unifies sensor access across different sensor configurations and technologies. Each car continuously determines relevant sensors for recording by requesting the vehicle-specific data requirements from the cloud platform. The recorded sensor readings are then transferred to the cloud and processed there. As a result, the readings are either streamed to the data consumer or persisted as files that map individual journeys from start to finish (trip files). The data consumer’s infrastructure can be connected directly to our cloud platform. The connection is established via a message-based interface that the consumer must provide. Our platform sends messages that include, for example, streaming data or notifications about newly created trip files. The use of our system is visualized in Figure 1.

### 3.1. GDPR Related Requirements

The GDPR distinguishes between personal data and non-personal data. Any processing of the former requires informed consent, which the data subject must give in advance [60]. Such consent includes a purpose limitation that also conclusively lists the parties involved in processing the data [61]. Additionally, the principle of data minimization must be applied. Therefore, only data indispensable for the stated purpose may be collected [13]. The GDPR does not regulate the processing of non-personal data. Consequently, a system that can legally process personal data is also allowed to process non-personal data.

The term personal data is to be understood very broadly. It refers not only to data that directly links a person, such as a name, but also to data with an indirect link to a person. Thus, a license plate is also considered personal data even though the processor might not be capable of looking up the owner. The mere possibility that such a lookup could be performed is sufficient for the classification as personal data [60]. The classification of a set of data into this binary scheme poses a considerable problem in practice [60]. Incorrect classifications can lead to severe penalties of up to €20 million or 4% of annual global turnover (whichever is higher) [62]. This is a strong incentive for companies to classify data as personal when in doubt.

The system we present is designed to meet the legal requirements for processing personal data (See Table 2). As such, we do not transmit any sensor data from a vehicle unless informed consent is given. The scope of data collection is determined individually for each vehicle by applying our demand-driven data acquisition strategy (See Section 3.2). In this way, we comply with the principle of data minimization and ensure that no data collection takes place without a purpose. Designing the system to handle personal data does not prevent its use for non-personal data, as explained in Section 3.8.

### 3.2. Demand-Driven Data Acquisition

**Figure 2:**
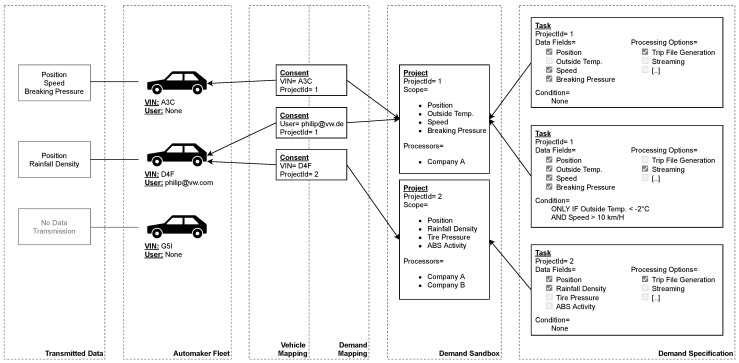
Application of the Demand-Driven Data Acquisition. The condition of one task does not apply. It is therefore not executed.

We have found that, in practice, an access pattern prevails in which individual data consumers want to access different sensors originating from different cars. There may be overlaps, i.e., multiple consumers can simultaneously access certain vehicles and sensors. The demand-driven data acquisition method we have developed can efficiently handle such patterns. It minimizes data collection for each vehicle individually. As a result, we only collect data required by the data consumers (*demand*) who obtained consent from an individual vehicle. An illustration of our applied method is presented in Figure 2.

Data consumers specify their *demands* within the context of so-called projects. Each project represents an independent data collection purpose within the meaning of the GDPR. It contains sensors that define the maximum scope of any data collection carried out through it. The *demand* is set separately from the project and can be continuously adjusted while being within the associated scope. The consent is directed at the project. Thus, a *demand* adjustment affects existing and new consents equally. The projects are immutable and represent reliable constraints on the associated *demand*.

The actual *demand* of a project is equal to the sum of its tasks. Each task contains a list of sensors to be acquired, an optional condition, and any processing-related options. Each vehicle is receiving all tasks for which project-related consent exists. The application deployed to the vehicles is executing them (See Section 4.4). Therefore, the condition is evaluated individually by each car and can include local sensor values, geographic restrictions, or time limits. A task can perform conditional data collection as it is only executed if the associated condition holds. Upon execution, the task-related sensors are recorded and transmitted with regard to the processing options: If streaming is activated, the recorded sensor readings are buffered locally for a shorter time, for example. Additionally, deduplication occurs; if several tasks contain a sensor, its readings are transmitted once.

Each consent links an individual car with a project. For this purpose, the vehicle can be addressed via its unique Vehicle Identification Number (VIN). Alternatively, indirect addressing can occur by utilizing the driver’s account used to log on to the car. Thus, the data of an individual driver can be collected across multiple vehicles. There can be multiple consents for each project and car.

### 3.3. Sensor Abstraction

A highly heterogeneous car landscape characterizes the automotive sector. For example, the Volkswagen Group is currently producing 391 different car models globally. They are built on top of distinct platforms that utilize different technologies. Every model can be configured to contain optional features upon ordering. These features may result in additional sensors being added to the car. There are at least two suppliers for every sensor. Each supplier can introduce custom characteristics into their products, like different data resolutions or access channels. Additionally, software updates may be performed on subsets of the overall fleet. Such an update can potentially alter sensor-specific data-access channels.

We have created an abstraction layer that reduces the variety of sensors to the individual *data fields* that can be read from a car. Consequently, our system only reveals high-level *data fields* such as “speed”. The underlying complex variety of different sensors is invisible to the data consumer. We map this variety via so-called instructions. For each *data field*, several instructions can exist. There is one for every distinct data-access channel that may exist. The application deployed to the vehicles can interpret such instructions to acquire the associated *data fields* from its sensors. It can thus also determine local sensor capacity by trying out the instructions: If the application can successfully execute at least one instruction of a *data field*, it is considered available.

The data-access channels have a multi-layered architecture: A custom application logic is implemented based on existing protocols such as WebSocket or MQTT. An example for such an implementation is the VIWI interface [63]. The instructions we utilize are implemented at the protocol layer. For each protocol, there is a dedicated instruction processing logic in the abstraction layer. The protocol-level implementation enables us to handle diverging application logic exclusively by adding additional instructions.

**Figure 3:**
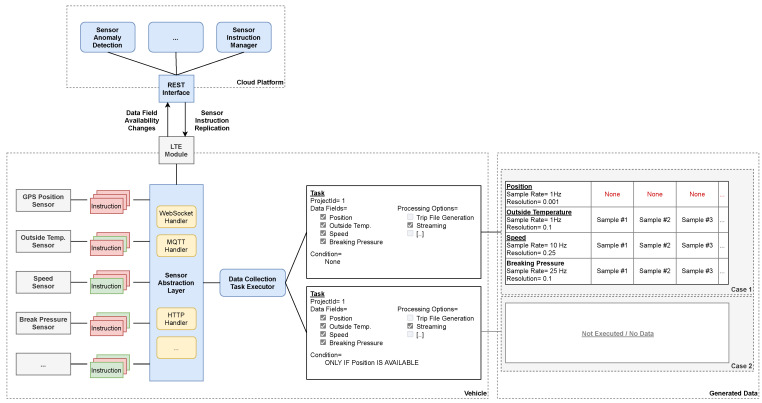
Usage of the sensor abstraction layer. Individual instructions are available for each sensor. Instructions that are compatible with the specific vehicle are marked in green, and incompatible instructions are marked in red. In the first case, as much data as possible is collected, resulting in a sparse dataset because a *data field* is not available. In the second case, no sensor readings are collected at all because the condition does not hold.

We have developed a replication mechanism for this purpose. It continuously transfers the available instructions to all vehicles (See Section 4.2). In addition, each vehicle reports local changes in *data field* availability. An anomaly detection evaluates these changes. We can thus detect breaking modifications to the data-access channels indirectly and restore compatibility by adding new instructions. Additionally, we can monitor the overall health of the system (See Figure 3).

The abstraction layer is also integrated with the task concept of the demand-driven data acquisition strategy. By default, a task yields a sparse dataset, i.e., if a *data field* is not available on a vehicle, the remaining *data fields* are still transmitted and made available to the data consumer. However, it is possible to include the availability of individual *data fields* into the condition. As a result, a task can be configured to yield a dense dataset as it is only executed if the given *data fields* are available on the vehicle (See Figure 3).

The sensor abstraction layer does not explicitly normalize local sensor peculiarities such as different data resolutions, sampling rates, or units of measurement. Instead, the sensor readings are transmitted unchanged in combination with metadata containing these peculiarities. Consequently, a data consumer can receive sensor data in different resolutions and sampling rates via a single task.

### 3.4. Data Transmission and Processing

The vehicle transmits recorded *data fields* as chunk sequences to the cloud platform. It generates an independent sequence for each trip, whose components are numbered consecutively. Each chunk can include time series for multiple *data fields*, and it is structured so that there are no dependencies on other chunks. Thus, if chunks are lost, the data loss is limited to those chunks. The vehicle knows which *data fields* are retrieved by tasks that have streaming enabled. It transmits the corresponding data more frequently serialized as separate chunks. A custom chunk compression strategy is applied as described in Section 4.5.

A three-phase protocol is employed to transmit each sequence to the cloud platform: First, the vehicle performs a trip initialization by submitting a unique TripID. Afterward, it transfers the chunks upon their generation. Finally, after the vehicle has transmitted all chunks, it sends a *commit* message that indicates the end of a trip. The *commit* message includes the TripID and the number of the final chunk.

**Figure 4:**
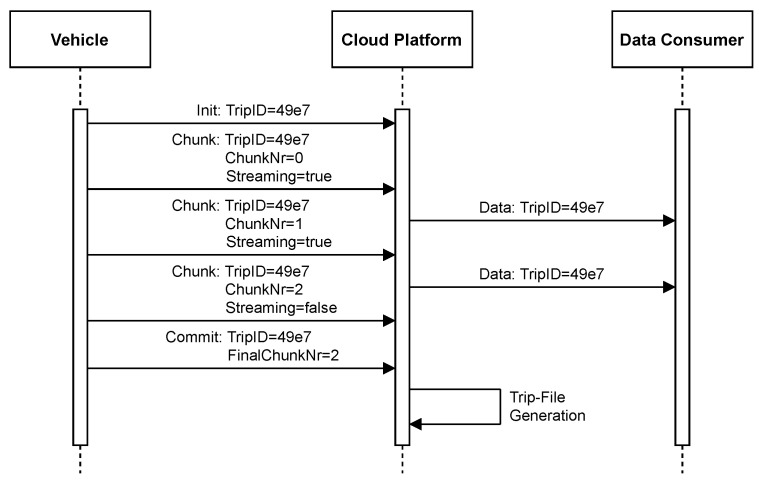
Transmission of a chunked trip sequence.

The cloud platform is storing all received chunks within a persistent buffer. Upon receiving the *commit* message, it performs an aggregation by merging all chunks of the trip. The resulting aggregate is split based on the contained tasks. A trip file is generated and stored for each task that has enabled persistence. Receiving a streaming chunk triggers its immediate processing: The contained data are transferred to the data consumers by utilizing endpoint messaging as described in the following subsection. The execution of a sequence transfer is shown in Figure 4.

Multiple persistence layers within the car ensure no data loss occurs if the car is turned off or has lost its internet connectivity, as described in Section 4.7. However, such a condition can last for an extended period, e.g., if the vehicle is in an underground car park. Thus, there is no way to ensure that the vehicle sends the *commit* message on time. We enforce a timeout to keep the buffer requirements predictable by avoiding indefinite buffering. When the timeout occurs, the cloud platform performs the aggregation, which results in trip files that contain a flag indicating their possible incompleteness. Otherwise, the completeness can be determined reliably with the final chunk number from the *commit* message. Tasks can be configured to discard incomplete files.

Trip files include a reference to the associated consents. If there is a consent revocation, then the corresponding files are automatically deleted from our platform. The data consumer will be notified of the revocation via endpoint messaging and can perform a local deletion by utilizing the embedded references.

### 3.5. Endpoint Messaging

**Figure 5:**
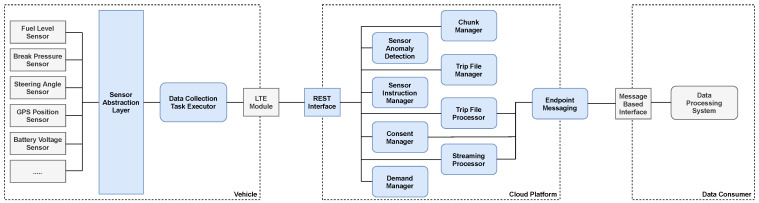
High-level architecture of the proposed system. Components that we have developed are coloured blue.

We assume that data consumers have automated systems for data processing. These systems can be connected to our cloud platform via a message-based interface (See Figure 5). Messages delivered through this interface can describe events such as new trip files or consent revocations. These messages only contain metadata that can be used, for example, to retrieve the actual trip file via our REST interface. In addition, we also use the message interface to deliver streaming data. Unlike trip files, it is limited in size because no aggregation is performed. Therefore, we embed streaming data directly within the message.

We use platform-independent Protocol Buffers to serialize the messages [64]. They enable us to support different transportation channels such as HTTPS, AWS SQS, or Google Pub/Sub. While the latter have their own authentication methods, the opposite is true for HTTPS. We utilize asymmetric cryptography to extend every message that is delivered via HTTPS with a signature. It can be verified by using our public keys.

The delivery of messages must be reliable, as the loss of individual messages can lead to a GDPR violation. If the data consumer is not notified of consent revocation, he may not fulfill his obligation to delete associated data. For this reason, we repeat delivery attempts until they finally succeed.

### 3.6. Vehicle Authentication

Requests originating from a vehicle are authenticated via signed tokens that contain the VIN and optionally the user id from the driver. A central Identity Provider (IDP) issues them. The vehicles have embedded certificates linked to their individual VIN. They utilize these certificates to obtain tokens from the IDP continuously. In addition, the driver can log in to the IDP via the vehicle user interface. Then, the IDP extends the token with the user id. Our cloud platform verifies these tokens and can thus provide the origin of individual data. Additionally, all communication between the vehicle and our cloud platform is encrypted by utilizing HTTPS.

### 3.7. Vehicle Simulators

**Figure 6:**
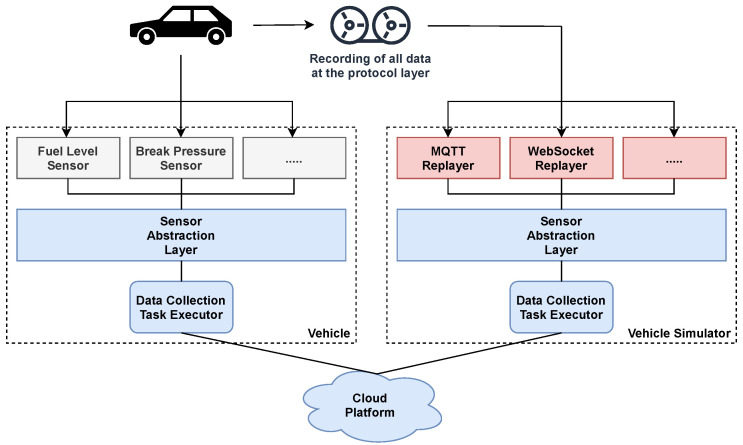
Implementation of a vehicle simulator. It executes the same software as the car. We provide simulated data-access channels that can replay arbitrary recordings from actual vehicles.

The system provides functionality to simulate active vehicles. It realizes such simulations by using recordings from real cars. The data contained therein were captured at the protocol layer and represents a complete snapshot of the data-access channels during a trip. These snapshots are replayed to create a virtualization of the data-access channels in the car. The same software components that are usually executed by the car are connected to them. The result is a realistic simulation covering all system features and offers the same data as the actual vehicle (See Figure 6).

Simulations of different vehicle models are provided by creating separate recordings for them. Model-specific characteristics, such as differences in the data-access channels, do not have to be explicitly mapped since the recordings are made at the protocol layer. We provide the recordings from different car models as a service.

The simulators are made available to the data consumers. They can thus develop their own data processing systems without needing any consent or own vehicles. We also utilize them to perform load tests (See Section 5) and functional tests.

### 3.8. Acquisition of Non-Personal Data

The presented system can also collect and process non-personal data for its data consumers. For this purpose, we utilize the mapping resulting from the consent: As soon as a consent object is present, an individual vehicle begins collecting and transmitting the associated *demand*. When dealing with non-personal data, we can create these objects without actual consent from the data subjects and utilize them to address individual vehicles for data collection. There are no additional legal requirements (See Section 3.1).

For practical use in the context of individual data consumers accessing subsets of the overall fleet, such addressing would have to be automated and intelligent: The intended fleet includes millions of vehicles spread across the globe. Therefore, we must select the vehicles that provide the most significant benefit for the goals of the data consumer. For example, if a data consumer wants to determine the most popular radio station in New York, vehicles in Brazil would be unsuitable. Economic efficiency correlates with selection accuracy since an incorrect selection generates costs that are not offset by benefits. Our system does not yet include such intelligent addressing. The selection must precede the task distribution because task conditions are not designed for this purpose. Using task conditions would require all non-personal data tasks to be executed on every vehicle. Consequently, we cannot provide access to non-personal data for arbitrary data consumers efficiently.

However, the system can still efficiently handle use cases that do not depend on such car selection optimization. Automakers have an interest in collecting data to track wear and tear on their vehicles. They are also interested in statistics on average consumption and emissions [21]. It is possible to acquire such data for each model individually: The automakers assign the VINs, which indicate the associated model. Thus, they can either create a consent object for all of them or perform sampling by only utilizing a subset of the models’ VINs.

## 4. Implementation

Our system is split into multiple components, which are distributed over different infrastructures (See Figure 5). Most of them are critical for the platform’s reliability and must be capable of handling high-frequency access patterns. We describe implementation details of selected components within this section.

### 4.1. Utilized Technology

The system components running on the vehicle were developed in Node.js without using an additional framework. They are being executed as a background application without having a user interface. Our cloud platform was developed in Python and uses the FastAPI framework [65]. The connection between the two systems is made via client libraries automatically generated from the Swagger definitions provided by FastAPI. In addition, we utilize Protocol Buffers for message and sensor data serialization [64]. Furthermore, the following services provided by Google Cloud Platform and their associated libraries are used:

- App Engine: A managed service to host web applications. It deploys a user-supplied application to proprietary virtual machines that spawn within seconds. Thus, it allows handling sudden spikes of traffic by adjusting the underlying servers just in time [66].
- Datastore: Managed NoSQL database that stores items with unique keys. Its underlying servers manage continuous subsets of the keyspace. Every item is limited to one update per second. It supports consistent queries and transactions spanning multiple operations [67].
- Cloud Tasks: Provides the capability to schedule asynchronous HTTP requests. It supports delayed executions and retries failed requests until they finally succeed. The requests are stored within queues [68].
- Pub/Sub: Asynchronous messaging service. Messages are published to topics that can have multiple subscriptions. Published messages are replicated to every subscription and will be delivered at least once [69].
- Compute Engine: A service that provides virtual servers and auto-scaling. The scaling can be tied to the in-flight messages of a Pub/Sub subscription [70].
- Cloud Storage: An object store that can persist unstructured blobs/objects within buckets. Events can be pushed to a Pub/Sub Topic [71].

### 4.2. Instruction Distribution

The instructions of the sensor abstraction layer must be eventually replicated to the connected cars (See Section 3.3). No inconsistencies may occur during this process, as this could cause *data fields* to become locally unavailable. This process is read-heavy and requires minimal writing: There are potentially millions of cars regularly querying for updates. On the contrary, we are adding new instructions very infrequently.

We utilize a replication strategy that relies on multiple Last-Writer-Wins Registers (LWW Registers), a known conflict-free replicated data type [72]. There is an individual register for each *data field* that contains all associated instructions. We eventually apply the final state of each register to the vehicles upon performing changes to them. For this purpose, we use an ordered sequence whose elements are the registers. The sorting order corresponds to the modification date of the registers, which is unique and consistent with causality. Thus, each element represents a savepoint from which the vehicle queries the following elements. If we make multiple changes to the same register, only the final state remains in the sequence. The vehicle can thus skip intermediate states within the replication process.

A distributed cache layer accelerates the sequence replication of the vehicles. They regularly query for elements to continue with the local sequence. In return, they receive the subsequent elements as a sorted list. The sorting property enables distributed caching that does not require synchronization. No inconsistency occurs when the last item of an ordered sequence is missing. A subsequent query will yield it as long as the cache eventually expires.

**Figure 7:**
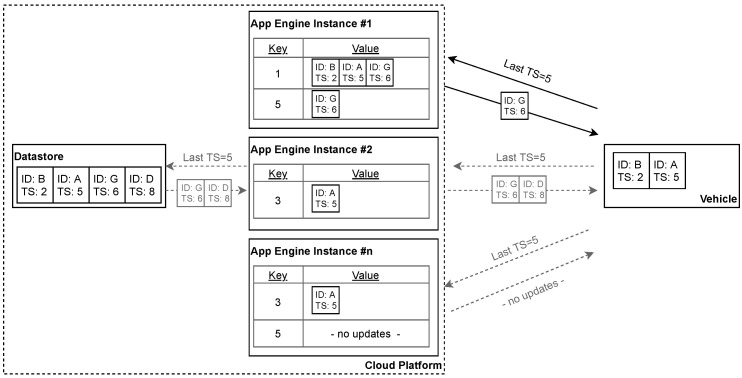
Sharding during instruction distribution. Datastore acts as single source of truth. App Engine instances cache queries locally. The timestamp submitted by the car acts as cache key and query parameter. The cache items can be out of sync as long as they represent an ordered subsequence and eventually expire (See Section 4.2).

We have implemented the instruction replication by utilizing Datastore and App Engine. The former acts as a reliable persistence layer. There is an item for every register, respectively, *data field* that contains all associated instructions. Each item has a timestamp property, and updates are performed in-place. The sequence is generated via a strongly consistent Datastore query [73] that performs sorting by utilizing the timestamp. The resulting sequences are cached on the individual App Engine instances and can thus be accessed without additional network calls (See Figure 7). The used timeout has a random component to avoid a cache stampede.

We use a monotonically increasing value as a timestamp. The system time is not suitable because we are not able to synchronize the clocks appropriately. Even if this were the case, a small risk of duplicate timestamps would remain. For this, we maintain a dedicated Datastore entry that acts as a counter. Each register change is a transaction that includes the counter and increments it to determine a new timestamp.

As a result, we have increased the read throughput by exploiting the auto-scaling capabilities of App Engine. However, the write throughput is limited to one *data field* update and creation per second due to the counter including transaction. As this guarantees consistency, we consider the restriction to be appropriate due to the rarity of instruction updates.

### 4.3. Consent Lookup

Each vehicle performs regular queries to the cloud platform to determine the relevant projects. They are determined by evaluating the active consents. For this purpose, each vehicle transmits its VIN and optionally a user id (identifiers). This operation is primarily read-heavy, but there cannot be any drawbacks on write performance. It must be possible to add and revoke the associated consents at a high frequency.

We store each consent as individual Datastore objects and maintain a reverse index for each identifier to enable querying by them. The access intensity to the individual objects tends to be equally distributed; consequently, we do not use a cache. Instead, we utilize the data distribution strategy of Datastore to balance the load evenly across the underlying server capacity. The assignment of an object to individual servers results from its key [67].

For each consent, we generate a random key with high entropy. The creation is done within a transaction to ensure that the key is unique. In addition, we maintain the reverse index within the transaction. For each identifier, there is an object storing all keys and project references of the associated consents. We derive the key of the reverse index object deterministically from the corresponding identifier. An index lookup is thus possible via a single get-operation.

The identifiers may represent monotonically increasing values or have a common prefix. For example, the first three characters of a VIN are identical for many vehicles because they contain a manufacturer’s abbreviation [74]. Such keys represent a Datastore anti-pattern causing a concentration of the associated objects on a small subset of the server capacity [75]. Therefore, we use a cryptographic hash function to create uniformly distributed prefixes for the identifiers. This additional prefix causes an even distribution of the associated objects.

We found that our implementation has few limitations. Each identifier generates approximately the same load, and we distribute the underlying objects evenly across the Datastore servers. We are thus not limited in the number of vehicles that query our platform simultaneously. There are also no limitations in terms of concurrent consent creations and revocations. However, there cannot be more than one consent revocation or creation per second for each distinct identifier. This limitation results from the transaction through which we maintain the reverse index. We consider the limitation to be appropriate.

### 4.4. Task Distribution

Each vehicle must determine the active tasks for the project references obtained through consent lookup (See Section 4.3). There is a continuous synchronization with the cloud platform so that task-related changes can be detected promptly. The process is predominantly reading-heavy. There is a substantial potential for contention if many vehicles are linked to the same project and thus query the same data.

A vehicle can be assigned to multiple projects, and each can contain multiple tasks. We have designed our process to avoid the synchronization of individual tasks. For this purpose, we have made the tasks immutable and manage a list of active tasks for each project. Thus, a task is modified by creating an updated task and deactivating the previous task. The vehicle synchronizes these references continuously and requests the associated tasks separately. Due to their immutability, tasks can be cached indefinitely by the vehicle and cloud platform.

We store the tasks as individual Datastore objects. In addition, there is a reverse index for each project that contains references to its active tasks. We store the reverse index as a separate object whose key is composed of the project key. This assignment enables an index-lookup via a single get-operation. When the data consumer creates or deactivates a task, the reverse index is updated via a transaction.

The reverse index includes a version property turning it to an LWW Register [72]. We can therefore cache the indexes on the individual App Engine instances without synchronizing them. The vehicle uses the version to ignore stale data. A short expiry interval ensures the timely propagation of updates.

By not synchronizing individual tasks, we reduce replication overhead. We can apply different caching strategies due to the separation of tasks and their activation properties. Immediate caching on App Engine instances automatically adjusts the read capacity to the current load. There is no limitation on overall projects and tasks. However, for each project, there can be only one task addition and deactivation per second. We consider this limitation to be appropriate.

### 4.5. Chunk Compression

Each vehicle continuously generates and transmits chunks. Under certain circumstances, persistence to a local storage medium occurs (See Section 3.4 and Section 4.7). Reducing the chunk size is thus a necessity. Local storage is limited and composed of eMMC memory. Writing large amounts of data may cause a memory failure [76]. In addition, multiple applications are consuming the limited internet uplink, and it may be degraded in rural areas [77]. Compressed chunks have less impact on other consumers, and their transmission is more likely to succeed on degraded networks. Furthermore, they improve economic efficiency as uploads from a vehicle are expensive.

We have developed a data preprocessing method that we combine with an existing compression method. As a result, we achieve a significantly higher compression ratio than either method can provide on its own.

The preprocessing procedure is lossless and targets the time series contained in a chunk. Its elements are composed of a limited set of primitives, and each has an associated timestamp. We convert all values to the smallest possible integers and apply variable-length integer encoding (VLI Encoding). There is a different conversion strategy for each primitive, as explained below.

1. Timestamp: The system does not have real-time capabilities and cannot perform measurements at exact intervals. In addition, most data-access channels only report measurements if they differ from the previous ones. Consequently, we cannot drop the timestamps in favor of storing the sequence interval length. However, many timestamps still represent recurring intervals with slight variations. They originate from sensors whose values changed with almost every measurement. For example, the engine speed most likely varies continuously, given a resolution of 1 RPM.
  In [46], the authors have found that Delta-Of-Delta (DOD) encoding is a good fit for timestamps with such characteristics. It enhances Delta encoding, which is a procedure that only keeps the first value of a sequence. The subsequent values are the delta to the predecessor (a[n]=a[n]−a[n−1]). In DOD encoding, the first value is unchanged, and the second is the delta to the first. Subsequent values are computed as follows: a[n]=(a[n]−a[n−1])−(a[n−1]−a[n−2]). Thus, they represent the delta of two deltas.
  Applying DOD encoding to the timestamps results in an average compression ratio of 3.66 (See Table 3).
2. Integer: We apply Delta encoding to integer values. The average compression ratio is 3.76. DOD encoding has not led to further improvements (See Table 3).
3. Float: We found that no sensor exploits the full precision of a float. Thus, we can perform a reversible conversion into integers (F2I) without information loss. The conversion is performed by shifting the decimal sign *n* places to the right ($float*10^{n}$) and cutting the remaining decimals. *n* is equivalent to the decimals of the sensor resolution. A resolution of 0.25 corresponds to an *n* of 2.
  Applying F2I with Delta- and VLI encoding results in an average compression ratio of 3.16 (See Table 3).
  We have dismissed two other approaches: The authors of [46] are utilizing XOR-based compression. Their method is optimized for repeating values and can encode them with a single bit. The reference data consist of 59% repetitions. Deducting them from their results gives a compression ratio of 2.09. Furthermore, we have validated the strategy presented by Lindstrom and Isenburg [45]. It relies on a predictor that benefits from repeated values as well. Applying it to our dataset resulted in an average compression ratio of 1.24.
4. String: The majority of string-producing sensors yield low cardinality ENUMs. We apply a dictionary compression scheme to them. The strings are stored once within the dictionary, and the time series only contains dictionary indexes. An average compression ratio of 3.53 was achieved (See Table 3).

We have applied multiple compression algorithms to the preprocessed data and found that preprocessing improves the compression ratio of every algorithm (See Table 4). We chose to use Gzip [47] due to its low resource requirements and wide availability. Our preprocessing achieves an average compression ratio of 2.99 (See Table 4). Applying Gzip afterward improves the ratio to 4.82 while using Gzip alone only results in a ratio of 1.84 (See Table 4).

### 4.6. Chunk Processing

Our system is processing chunks to generate trip files and perform near real-time streaming. Both operations have different service level requirements. Trip file generation is not a time-critical operation, and thus large fluctuations in the execution time are tolerable. For streaming, processing must be as fast as possible, and prolonged outages are not acceptable. Therefore, we operate separated infrastructures for each operation, aligned to the appropriate service levels. This separation allows us to make data processing more cost-effective.

**Figure 8:**
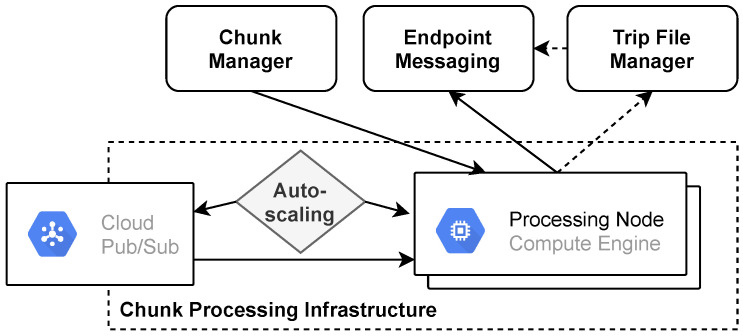
A message-based chunk processing infrastructure architecture: There is a pool of stateless processing nodes to handle the messages. Each message represents an executable job. We use this architecture to perform streaming or to generate trip files.

Both infrastructures use the same message-based architecture (See Figure 8). In this architecture, each message represents an executable job that is self-contained and idempotent. The messages are consumed and processed by a server pool whose capacity is automatically adjusted. There is a persistent message broker that handles job scheduling and thus decouples scheduling from execution. The decoupling enables us to prevent the overloading of individual servers. Each server will only execute a limited number of jobs in parallel. If job creation frequency exceeds the pool’s processing capacity, the broker will queue the excess. Job scheduling is possible even if there are no servers in the pool.

We adjust the processing pool capacity based on the number of messages scheduled for execution and currently being executed. For this purpose, we determine how many servers are required to process these messages in parallel. In addition, we grant each server a backlog that affects scaling aggressiveness (See Equation (1)). A larger backlog tends to increase average processing time but suppresses erratic upscaling.

(1) required_servers=messages/per_server_limit*backlog_modifier

The trip file creation infrastructure utilizes preemptible Compute Engine instances for message processing. These instances provide a cost advantage but have a limited lifetime, and the cloud provider can terminate them at any time. In addition, there may be periods when preemptible instances are not available due to lack of supply [78]. Furthermore, we use a higher backlog modifier and thus accept longer delays in execution. For the streaming infrastructure, we use regular Compute Engine instances and a reduced backlog modifier.

The vehicle performs a chunk transmission in two phases. Initially, it sends the chunk associated metadata and receives a signed Cloud Storage URL in response. The chunk is then uploaded via the received URL. We create a Datastore object for each trip to store the associated chunk metadata and Cloud Storage references. Its key is composed of the trip identifier, a random value that ensures even distribution across the Datastore keyspace.

Our cloud platform schedules the trip file creation upon receiving a *commit* message from the vehicle. For this purpose, we load the trip-associated Datastore object to access the Cloud Storage references and metadata. These fields are used to create a message that we send to the message broker of the corresponding infrastructure. During processing, a server from the pool loads all trip chunks and merges them. It creates a separate file for each contained task and forwards it to another system for storage (See Figure 8).

The upload of a streaming chunk immediately triggers its processing. The Cloud Storage bucket sends a message upon receiving the chunk. A random server from the processing pool loads the chunk and forwards the data using endpoint messaging.

Chunk receive capacity is limited to one per trip and second. This limitation originates from the single Datastore item utilized to store trip-related chunk metadata and Cloud Storage references. There is no limitation on concurrent trips due to the randomized Datastore keys and the fact that the throughput of Cloud Storage and Pub/Sub is not limited.

### 4.7. Chunk Processing Fault Tolerance

Chunk processing is a distributed transaction spanning multiple system boundaries. Individual failures may not result in data loss or a corrupted state. We have developed a transaction procedure that relies on passing multiple savepoints sequentially. Each savepoint represents a self-contained and consistent state. Thus, reaching a new savepoint enables the deletion of the previous state. Operations to reach a new savepoint are designed to be idempotent and repeated until they eventually succeed.

The vehicles transmit their data via a three-phase protocol. In this protocol, the sequence of operations is essential. For example, a *commit* may only occur when all chunks are transmitted (See Section 3.4). The vehicle manages the corresponding operations in a queue and executes them sequentially using the first-in-first-out strategy. An operation is only removed from the queue after its successful execution. If an error occurs, the vehicle delays the next iteration by applying an exponential backoff. The vehicle persists the queue to a local storage device upon shutdown. It restores the queue and resumes execution when the car is started again.

The cloud platform receives the vehicle data idempotently and performs deduplication for this purpose. We use deterministic key generation to ensure that repeated invocations of the same operation will affect the same objects within our system. The initialization of a trip results in creating a cloud task, which eventually executes the timeout. We name the task by combining the trip identifier and the VIN. Cloud Tasks performs deduplication based on the task name, so reinitialization does not yield another timeout task [68].

Repeated chunk transmissions are also directed to the same Cloud Storage object. Additionally, we only store distinct references within the associated reverse index. Thus, we can avoid a continuous object expansion caused by a repeated transmission error. The vehicle only considers a *commit* to be successful if the cloud platform has created and persisted a message to initiate the trip file processing. Otherwise, it repeats the *commit* as already described.

By persisting the chunk processing message, we can guarantee its eventual processing. We obtain this guarantee by utilizing the Pub/Sub message redelivery strategy. Pub/Sub requires an explicit ACK for each message. Otherwise, it applies an exponential backoff and schedules the message for redelivery [69]. We ACK a message only in case of successful execution. The execution itself is designed to be idempotent. Thus, it does not alter or delete the associated chunks. Chunk deletion is carried out by expiry instead, for which we use a multiple of the Pub/Sub message retention time. Chunk processing represents the final savepoint. It encapsulates external calls, such as endpoint messaging. If these calls fail, the whole processing operation is eventually re-executed.

The trip file generation infrastructure performs deduplication as part of the processing. Immediately after a server has received a job, it checks whether a Datastore object exists whose key is composed of the trip identifier and the VIN. If this object exists, it skips the job. Otherwise, it executes the job and creates the object after successful execution. There is no mutex to prevent this logic from running in parallel so that race conditions can occur. In reality, most duplicate calls are filtered. The applied logic does not limit processing capacity, as the trip identifier is a random value with high entropy. Thus, the used objects are evenly distributed within Datastore.

As a result, the savepoints ensure that a wide variety of system components can fail without causing any data loss. However, chunk and message expiry require us to act timely on processing related outages. We must address them before expiration to avoid data loss. Thus, we continuously monitor related Pub/Sub metrics such as backlog size and oldest message age.

### 4.8. Trip-File Management

**Figure 9:**
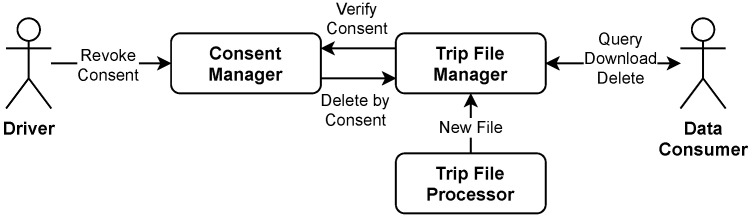
Interactions of the Trip File Manager with other system components or stakeholders. They may be performed concurrently and have the potential to cause inconsistencies.

The Trip File Manager is responsible for persisting trip files and providing access to them. It stores them on Cloud Storage and manages their metadata as Datastore objects. There can be parallel and duplicate calls to the provided functionality (See Figure 9). Thus, there is a high risk of inconsistencies. Any inconsistency may constitute a data protection incident in the context of the GDPR and can have serious consequences. To prevent inconsistencies, we have adjusted the order of our operations and use Cloud Tasks to ensure their eventual execution. The following causes for inconsistency were identified and addressed by us:

1. The Trip File Manager may not accept files associated with revoked consents.
2. The revocation of consent must eventually result in the removal of all associated files without any leftovers.
3. A previously deleted file must not become available after its repeated submission.

When a data subject revokes consent, we first block the creation of additional files before initiating the deletion of existing files. This order enables a one-time sequential deletion. Upon receiving a revocation request, we create a cloud task that encapsulates both operations and eventually executes them in the given order. We perform the blocking by adding a revocation flag to the Datastore object storing the consent. The deletion is initiated by creating another task, which then performs the same.

The deletion task executes a Datastore query, which targets all files associated with the consent. It then fetches only a subset of the result and creates another task containing the query’s cursor. Afterward, it sequentially deletes the individual files contained in the subset. The additional task does the same but utilizes the cursor to skip previous files. We thus distribute the deletion across multiple servers, each handling a small fraction of the associated files.

We create a cloud task for each trip file to be deleted. First, it turns the associated Datastore object into a tombstone by adding a flag. The object thus becomes invisible to user-facing queries, and the associated file can no longer be accessed. In addition, the tombstone blocks the re-creation of the associated file. The task deletes the files afterward from Cloud Storage.

The trip file manager rejects creating a trip file if an associated consent was revoked or a tombstone is present. It initially writes the file to Cloud Storage. Afterward, it performs a Datastore transaction fetching the consent objects and a potentially existing metadata object. If either a consent was revoked or a tombstoned metadata object exists, it cancels the transaction and deletes the associated Cloud Storage item. Otherwise, we create the metadata object within the same transaction. We ensure idempotency by creating deterministic Datastore keys and Cloud Storage names. The operation is only used by the trip file processor and thus retried until it eventually succeeds (See Section 4.7).

Preventing the identified inconsistencies has resulted in a limited trip file creation frequency for each consent. This limitation originates from the transaction that includes the metadata object and all associated consents. We have deemed this limitation acceptable: A consent usually relates to a single car, resulting in a limited creation frequency on its own.

## 5. Performance Evaluation

We have conducted a performance evaluation to assess the scaling capabilities of our system. For this purpose, we have simulated more than 200,000 vehicles simultaneously transmitting data to our system. As cars usually idle 95% of the time [79], this simulation is representative of a much larger fleet in reality. The scope of data collection thus corresponds to what commercially available products in the field of connected vehicles also have to achieve.

The performance evaluation is carried out by using real vehicle data: We have performed a 15-min drive with a Volkswagen T6.1 and recorded all data provided by the vehicle’s data-access channels. The resulting recording was used to generate virtual vehicles using our simulators, which realistically repeat the drive (See Section 3.7).

We aim to answer the following questions via the performance evaluation:

1. Can the system react to elevated load with an automated increase in server capacity?
2. Is the increased server capacity able to keep processing time constant?
3. Can the system respond to reduced load by automatically reducing server capacity?

### 5.1. Setup

**Figure 10:**
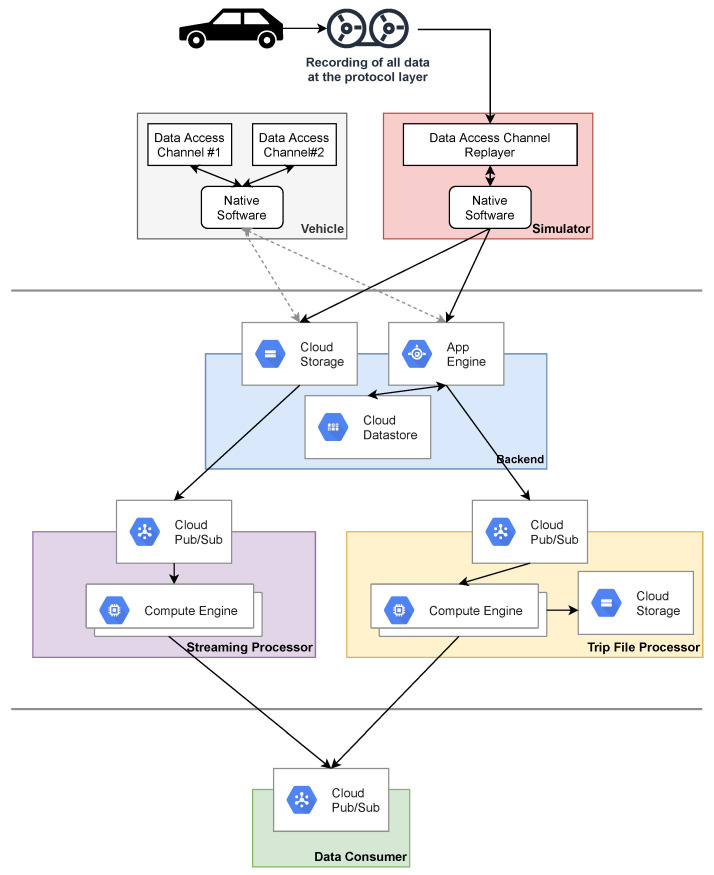
Setup of the performance evaluation: Using our simulators, we generate the same load as regular vehicles. The load is processed by three different infrastructures, for which we perform separate monitoring.

We have configured our system to make use of all its components. Therefore, we have created a project containing a single task that has enabled streaming and trip file generation. Since there are separate infrastructures for each of these kinds of data processing (See Figure 10), we can thus utilize both (See Section 4.6). In addition, we make use of endpoint messaging by configuring the project and task to use a Google Pub/Sub endpoint to receive the streaming data and trip file notifications (See Section 3.5). Furthermore, the simulators do not have any instructions pre-installed and must perform a complete instruction replication on startup. The other components, such as vehicle authentication or chunk compression, are integral to data processing and do not require separate configuration.

The cloud system is distributed across three infrastructures (See Figure 10): There are separate message based infrastructures to handle streaming and trip file processing. Furthermore, there is a REST backend providing all remaining functionality which is served via App Engine.

Streaming and trip file infrastructures are configured to use e2-highcpu-2 Compute Engine instances. Each instance may process up to 20 streaming chunks or 17 trip aggregations in parallel. We have set the backlog modifiers to 30 and 368 for streaming and trip processing, respectively (See Section 4.6). The backend is configured to use F1 instances with a target CPU utilization of 70% and a limit of 15 concurrent requests.

Performance monitoring is conducted by using Cloud Monitoring [80]. We record the number of active vehicles and the server count of each infrastructure. For the backend, we record the average request latency and request volume. Furthermore, we capture the average processing time and the number of unprocessed messages for the message-based infrastructures.

### 5.2. Results

**Figure 11:**
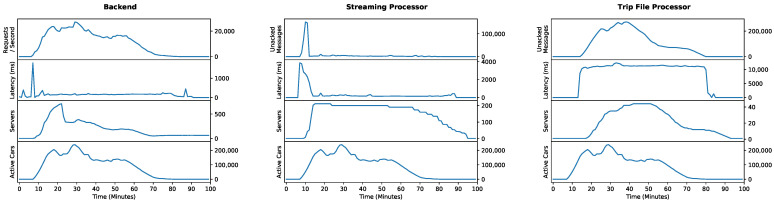
Results of a performance evaluation that was conducted by simulating over 200,000 concurrent cars. Performance metrics have been recorded for each of our infrastructures. It can be seen that the applied load causes an initial latency spike on the App Engine and Streaming infrastructure. This results from the rapidly increasing load for which insufficient capacity is available at this moment. The latency returns to normal after the capacity was automatically increased. It remains continuously low and thus enables time critical processing operations even at peak load. The latency of the Trip File Processor is elevated but stays on the same level. Its operations are not considered to be time critical. Thus, we have applied a less aggressive scaling strategy.

We have found that the first and third questions can be answered positively. All monitored infrastructures were able to increase server capacity in response to the increased load. In addition, server capacity was reduced in response to the drop in load (See Figure 11).

The second question can also be answered positively. There were initial spikes in latency on the backend and streaming infrastructure. They were caused by insufficient server capacity to handle the rapidly increasing load. After an automated adjustment, the latency remained continuously low even at peak load. At that time, the average streaming processing latency was 269 ms, while the backend had an average request latency of 190 ms. Trip file processing latency was consistently higher due to a less aggressive scaling strategy. This result is anticipated, as we consider the creation of trip files a time-insensitive operation (See Section 4.6).

A summary of the measured latencies can be found in Table 5.

## 6. Discussion

### 6.1. Demand-Driven Data Acquisition

The presented demand-driven data acquisition makes large fleets accessible to individual data consumers more efficiently than previous methods. Instead of acquiring a static set of sensors from each vehicle, such as [16,18,19,20,21,22,23,24,25,30,31,32], we minimize the scope of data acquisition for each vehicle individually. This optimization facilitates access to subsets of the total fleet and sensor capacity, as the remaining data do not need to be transmitted and processed. Thus, operational costs no longer depend primarily on fleet size but rather on the actual data consumption of the data consumers. This is not the case with any of the other works.

Minimizing the scope of data acquisition is based on the *demand* specified by the data consumers. For this purpose, they manage so-called tasks. These tasks can include conditions that are evaluated locally on the vehicles, enabling conditional data collection. In addition, the options for data processing are part of each task. By coupling these options to the condition, dynamic choice of data processing is possible. Some of the presented systems can also evaluate conditions. However, they evaluate it on their backends and utilize the condition to yield events [31,81]. None of the other systems can make data collection and data processing dependent on a condition evaluated on the individual vehicles.

We can process personal data in a GDPR compliant manner. The vehicles do not transmit any personal data without the prior consent of the data subject. Many of the present works do not consider consent handling, e.g., [16,19,20,21,26,28,29]. The authors of [17] utilize a smartphone app that asks for permission before processing data. Some works acquire data without prior consent and request consent to access the data already stored on their platforms [22,23]. By separating consent and *demand*, we enable the data consumers to continuously adjust their data collection within the boundaries of the consent—even after it was given. In addition, we can address individual vehicles for data acquisition by utilizing the identity signed on to the car. No other system enables data consumers to adjust their collection of personal data for existing consents or tracking data subjects across different vehicles.

Furthermore, the presented system is a suitable low-level architecture to acquire and process non-personal data. The consent objects can be utilized to address individual vehicles to start data acquisition on them. All benefits of the system also apply to collecting non-personal data, e.g., the demand-driven data acquisition, scalability, abstraction layer, or data compression.

### 6.2. Abstraction Layer

The presented system has an abstraction layer to integrate a heterogeneous fleet through a single interface. There is an instruction for each sensor particularity existing in the fleet. The vehicles utilize them to self-determine their sensor capabilities and retrieve the associated data. In addition, conditional task execution can be performed depending on sensor availability. Many of the presented works do not consider the need for an abstraction layer [16,17,18,19,20,25,26,27,28,55]. The authors of [29] would like to establish new technical standards to provide interoperability across different vehicles. In [23], the authors propose a standard based on a common data format: Automakers should adhere to the format and use it to submit data to a data marketplace. Another work is utilizing a mapping table to map varying OBD data IDs with a common natural language identifier such as “SpeedKmHr” [22]. The authors of [21] make use of OBD discovery in order to determine sensor availability and report it to their platform.

The *data fields* exposed by our abstraction layer are similar to *virtual sensors*, which are software components that process and make data available from physical sensors or other software components [82]. There are implementations of *virtual sensors* that abstract sensor access via a unified interface. However, the software components of these implementations are strongly coupled to specific sensors and communicate directly with them [83,84]. Our abstraction layer is more decoupled from actual sensors and requires little to no modifications when integrating a new vehicle model that has new sensors. Compatibility can be retrofitted via the replicated instructions since the underlying protocols usually remain static. Furthermore, we enhance the provided data with metadata from the originating sensor as already suggested by [82].

### 6.3. Data Compression

We developed a method for data preprocessing and combined it with an existing compression algorithm. This combination resulted in a significant reduction in the size of transmitted data without any loss of information. Many other works do not consider compression [16,19,20,21,24,25,26,27,28,29,55]. In [18], the authors report using a lossless trajectory compression method. Another work is utilizing downsampling and histograms to reduce transmission size [23]. The authors of [54] also propose a lossy compression that restricts data transmission to anomalies detected locally on the vehicle. Finally, the authors of [22] mention using a proprietary message format to minimize the volume of transmitted data but do not elaborate on this issue. We have also compared two stand-alone methods for the compression of time series data and found that they are not efficient within our domain (See Section 4.5).

### 6.4. Data Processing

Our system can record all data generated during a trip and provide it to the data consumer. By using sequence numbers, we can validate the completeness of trip-related data. Some systems provide the latest value for each sensor and therefore require the data consumer to poll, which can cause value changes to be missed [36,37,38]. Other systems have a streaming interface to push value changes directly to the data consumer. However, the consumer cannot determine whether all data of a trip were received [33,34,35]. In addition, some platforms store every received sensor value but cannot determine the end of a trip. Therefore, they cannot reliably generate trip aggregates [18,19,21,22,23]. The authors of [23] have also mentioned the use of sequence numbers to determine the completeness and correctness of data. However, they do not describe the consequence of missing sequence numbers. Finally, Refs. [18,22] have identified "data loss" as a common issue within this area.

We have implemented the processing of each trip’s data as independent and fault-tolerant transactions. Multiple savepoints enable operations to be retried upon errors until eventual success. This implementation prevents data loss during server failures, connection problems, or a car shutdown. The authors of [22,27] utilize a vehicle-side buffer to repeat transmissions in case of errors. Only [22] uses a persistent buffer that can survive a shutdown. The other works do not describe strategies to avoid data loss in case of transmission errors [16,18,19,20,21,23,24,25,26,28,29,55]. Our implementation moves beyond preventing transmission errors and extends to the subsequent data processing.

Our implementation achieves scalability through sharding. For this purpose, we exploit the internal data distribution of the utilized cloud technologies like Datastore. Our servers are stateless, except for caching. We can thus distribute requests evenly and randomly among them and adjust server count on demand. The statelessness also enables us to use cost-effective preemptible instances for trip file processing. In [26], the author has identified the acquisition and processing of vehicle data as a scaling issue. He is suggesting the use of cloud technology. The authors of [22] utilize a SQL Database to store the acquired data and perform partitioning by creating a separate table for every car. The database is hosted on a cloud server. Two other works also state to use cloud servers [19,24]. The authors of [16,18] make use of different storage systems. The most requested data are stored in memory, while pre-processed data are stored in a relational database, and archival takes place in a NoSQL database. Most works do not consider scalability [20,23,25,27,28,29,55]. Furthermore, none of the systems can perform automatic scaling by adjusting its server count. In addition, no system does utilize preemptible instances.

### 6.5. Data Authenticity

We can verify the authenticity of the received data because the vehicles authenticate themselves via an embedded certificate. In addition, the driver can log in to his car and thus effectively authenticates against our system. Many of the presented works do not include procedures to verify the authenticity of received data ([16,18,19,20,21,22,23,26,27,28,55]). The authors of [29] also identified the need to verify the vehicle identity but do not specify a method for it. Another work also proposes to issue a certificate to each car for authentication [85].

### 6.6. Performance Evaluation

We performed a simulation by virtualizing the data-access channels with a recording from an actual vehicle. By running our car application combined with the virtualized access channels, we could simulate over 200,000 concurrent vehicles. The average processing time of streaming data is 269 ms at peak load. In [18], the authors injected a historical dataset into their system. It can handle over 30,000 concurrent vehicles and has an average processing time of fewer than 2 s. The authors of [59] evaluated the processing performance of the commercial AutoPi Cloud and measured an average processing and transmission time of 3 s. Another system handles data from 5500 vehicles during production [24]. The other presented systems do not perform a performance evaluation [19,21,22,23,25].

## 7. Conclusions

In this work, we present a system that enables the opening of large fleets of heterogeneous vehicles for individual data consumers. We have found that, in practice, different data consumers have diverging data requirements in terms of required sensors. In order to handle such access patterns efficiently, we have developed an original demand-driven data acquisition strategy. It reduces data acquisition for each vehicle individually to only transmit data required by the data consumers (*demand*). Furthermore, we map individual *demands* to individual vehicles; thus, each car potentially transmits different sensors. Our design enables *demand* adjustments even after the data subjects consent by directing the consents to an immutable maximum collection scope (project). The data consumers express their *demand* as a set of tasks that include locally evaluated conditions and processing options.

Our implementation consists of a cloud platform and a software component we have integrated natively with a current vehicle platform of the Volkswagen Group. Automakers usually operate highly heterogeneous fleets, for which we can provide access via a single interface. For this purpose, we have developed an abstraction layer based on the distinct *data fields* vehicles can provide. Data transmitted by the vehicles is compressed by combining custom data preprocessing with an existing generalized compression algorithm. A validation with sensor data from an actual vehicle has shown this strategy to be significantly more efficient than existing generalized compression algorithms alone. The cloud platform provides data streaming or performs a recording of individual trips. We have explicitly partitioned the data flows within the platform to distribute them evenly across multiple systems. This partitioning scheme enables us to apply horizontal scaling, which we have implemented as an automated operation: Servers are added or removed based on the current system load. The processing of vehicle data is implemented as distributed transactions. We persist our data on redundant public cloud services; our servers are stateless and can fail without data loss. This statelessness allows us to make use of preemptible instances with low availability for selected data processing jobs. The overall system performance was evaluated by simulating data acquisition from over 200,000 cars simultaneously.

By integrating our system into a current vehicle platform of the Volkswagen Group, we have demonstrated the capabilities of modern mainstream cars. We have thus shown that it is possible to transform an automaker’s fleet into a globally distributed sensor network by deploying software. However, the resulting architecture is not limited to Volkswagen vehicles. It is essentially a system to acquire data from large distributed and heterogeneous multi-sensor networks. As such, other automakers or operators of large sensor networks may also utilize it.

We believe there is great potential in extending this work with an intelligent vehicle selection to improve efficiency for collecting non-personal data (See Section 3.8). Personal data also contain information that is not related to the identity of the data subject. Integration of differential privacy [86,87] or local differential privacy [88] into the proposed system would make it available while preserving user privacy. Furthermore, integrating blockchain into consent handling could enhance trackability and verifiability of personal data processing [89].

## Figures and Tables

**Table 1:** Overview of closely related data acquisition systems regarding selected features, including commercial closed-source products, which are marked with an asterisk. Features that are not evident from their public documentations are marked with a dash. There are also architectural concepts that assume a native integration but have not integrated their architecture into a vehicle yet. Some systems include programmable hardware; in this case, we only consider software and cloud platforms provided by the manufacturer [14,15].

	Feature	Demand Driven Data Acquisition ${}^{1}$	Multi Tenancy ${}^{2}$	Vehicle External Data Storage ${}^{3}$	Vehicle External Data Streaming ${}^{4}$	Conditiona Data Acquisition ${}^{5}$	Individual Vehicle Addressing ${}^{6}$	Consistency Guarantees ${}^{7}$	Data Compression ${}^{8}$	Native Integration ${}^{9}$
Work										
[16]		No	Yes	Yes	Yes	No	No	No	No	No
[17]		No	No	No	No	Yes	No	No	No	No
[18]		No	No	Yes	Yes	No	No	No	Yes	No
[19]		No	No	Yes	No	No	No	No	No	No
[20]		No	No	Yes	No	No	No	No	No	No
[21]		No	No	Yes	No	No	No	No	No	No
[22]		No	Yes	Yes	No	No	No	No	Yes	No
[23]		No	Yes	Yes	Yes	No	No	No	Yes	Yes
[24]		No	Yes	Yes	No	No	No	No	No	No
[25]		No	Yes	Yes	No	No	No	No	No	No
[26]		No	Yes	Yes	No	No	No	No	No	Assumed
[27]		No	Yes	No	Yes	No	No	No	No	Assumed
[28]		No	Yes	Yes	No	No	Yes	No	No	Assumed
[29]		No	No	Yes	No	Yes	Yes	No	No	Assumed
[30] (*)		No	Yes	Yes	No	No	No	-	-	No
[31] (*)		No	Yes	Yes	No	No	No	-	-	No
[32] (*)		No	Yes	Yes	Yes	No	No	-	-	No
[33] (*)		No	Yes	Yes	Yes	No	-	-	-	Yes
[34] (*)		No	Yes	Yes	Yes	No	-	-	-	Yes
[35] (*)		No	Yes	Yes	Yes	No	-	-	-	Yes
[36] (*)		No	Yes	Yes	No	No	-	-	-	Yes
[37] (*)		No	Yes	Yes	Yes	No	-	-	-	Yes
[38] (*)		No	Yes	-	-	No	-	-	-	Yes
[39] (*)		No	Yes	Yes	Yes	No	-	-	-	Yes
[14]		No	Yes	Yes	No	-	Yes	No	-	No
[15]		No	No	No	No	No	Yes	No	No	No
**Our Proposal**		**Yes**	**Yes**	**Yes**	**Yes**	**Yes**	**Yes**	**Yes**	**Yes**	**Yes**

^1^ Vehicle-specific scope reduction of transmitted sensors based on data-requirements of data consumers (See Section 3.2). ^2^ Functionality to let several independent parties work with the system, providing each party potentially different data. ^3^ Data can be transferred from the vehicle to an external system and persisted there. ^4^ The external system continuously provides the received sensor data to other systems via streaming. ^5^ Data transmission can be made dependent on a condition evaluated on each vehicle locally. ^6^ It is possible to collect data from a specific car within a fleet without collecting data from the other cars. ^7^ The system can collect consistent sensor time series and detect inconsistencies if necessary. ^8^ The vehicle compresses data before transmitting it to an external system. ^9^ The system is a native component of a car and does not require any additional hardware.

**Table 2:** Requirements for the processing of personal data derived from the GDPR.

Requirement	Realization	Section
Personal data may only be processed if prior consent is given.	The data collection software natively integrated with the vehicle does not collect and transmit any data by default. An individual vehicle only performs data collection if a consent object exists within our system associated with the vehicle.	Section 3.2
The collection of personal data may not exceed the scope of the associated consent.	The data subject consents to grant the data consumer access to an immutable set of sensors (project). Subsequent data acquisition by the data consumer can not exceed this scope.	Section 3.2
If consent was revoked, all associated data must be deleted from our system.	Personal data stored on our system is linked to the associated consent. If consent is revoked, we automatically delete the associated data.	Section 4.8
If consent was revoked, all associated data must be deleted from the infrastructure of the data consumer.	We notify the data consumer about the revocation via a message-based interface. The data provided to him always references the associated consent. Thus, he can carry out a targeted deletion on his infrastructure.	Section 3.5
If consent was revoked, associated data acquisition must stop; no further data may be collected.	Each vehicle communicates continuously with our cloud platform to stop individual data acquisitions if associated consent is revoked. The cloud platform will discard data the vehicle transferred before the revocation was propagated upon receipt.	Section 4.3 and Section 4.8
No more data may be collected than necessary (data minimization).	Each vehicle only transmits sensors required to fulfill the data *demand* of the data consumers for which associated consent exists. Data consumers specify *demand* according to their needs and can adjust the scope of data acquisition even after consent is granted.	Section 3.2

**Table 3:** Performance of the preprocessing methods.

Primitive	Preprocessing	Average Size	Ratio
Timestamp ^1^	None	5563 Byte	-
	Delta + VLI	1748 Byte	3.18
	DOD + VLI	1518 Byte	3.66
Integer ^2^	None	1069 Byte	-
	VLI	445 Byte	2.40
	Delta + VLI	284 Byte	3.76
	DOD + VLI	284 Byte	3.76
Float ^3^	None	7050 Byte	-
	F2I + VLI	3239 Byte	2.18
	F2I + Delta + VLI	2228 Byte	3.16
	F2I + DOD + VLI	2243 Byte	3.14
String ^4^	None	120 Byte	-
	Dictionary + VLI	34 Byte	3.53

The results have been computed from datasets acquired from a real vehicle that was driven on a public street. ^1^ 291 sequences; 39 sensors; 269,678 samples. ^2^ 109 sequences; 12 sensors; 29,143 samples. ^3^ 137 sequences; 11 sensors; 241,231 samples. ^4^ 127 sequences; 17 sensors; 2330 samples

**Table 4:** Combining our preprocessing strategy with generalized compression algorithms.

	Preprocessing Applied		
Algorithm	No (Ratio)	Yes (Ratio)	Time (ms)
None	1.00	2.99	-
Gzip [47]	1.84	4.82	38.08
bzip2 [48]	1.87	5.05	21.71
LZMA [49]	2.68	5.36	121.32
LZ4 [50]	1.43	4.05	82.00
Brotli [51]	2.60	5.59	762.41
zstd [52]	1.97	5.01	377.04
zlib [53]	1.84	4.83	38.92

The compression ratios are related to the average size of a chunk (182 kB). The underlying dataset was acquired from a real vehicle. It contains 589,904 samples from 39 different sensors.

**Table 5:** The latency of the individual infrastructures during the experiment.

	Latency (ms)			
Infrastructure	Mean	99th Percentile	95th Percentile	STD. Dev.
Backend	221	471	230	370
Streaming	192	1220	350	263
Trip Files	11329	14020	12997	880
